# Synthesis and Characterization of Flower-like Carbon-encapsulated Fe-C Nanoparticles for Application as Adsorbing Material

**DOI:** 10.3390/ma12050829

**Published:** 2019-03-12

**Authors:** Lixin Zhao, Xibin Dai, Baoe Li, Hongshui Wang, Haipeng Li, Chunyong Liang

**Affiliations:** 1 School of Materials Science and Engineering, Hebei University of Technology, Tianjin 300130, China; zhaolixin0410@163.com (L.Z.); dainothingpossible@163.com (X.D.); libaoe@hotmail.com (B.L.); kingflood@163.com (H.W.); 2 Research Institute for Energy Equipment Materials, Hebei University of Technology, Tianjin 300130, China

**Keywords:** carbon-encapsulated magnetic nanoparticles, chemical vapor deposition, magnetic property, adsorptive property

## Abstract

Carbon-encapsulated Fe-C (Fe-C@C) nanoparticles with a divergently flower-like morphology were successfully synthesized for application as an adsorbing material by using freeze-drying and chemical vapor deposition (CVD) methods. The Fe metallic source was first loaded onto a sodium chloride (NaCl) supporter via freeze-drying to obtain the Fe/NaCl composite powder. Then, Fe-C@C nanoparticles were synthesized in the temperature range of 300–450 °C via CVD of acetylene in the Fe/NaCl composite powder using Fe nanoparticles as catalysts and NaCl as supporters. Because the NaCl supporter is water-soluble, the synthesized Fe-C@C nanoparticles were easy to purify, and a high purity was obtained by simple washing and centrifugation. The optimal Fe-C@C nanoparticles, synthesized at 400 °C, possessed a unique divergently flower-like structure and a high specific surface area of 169.4 m^2^/g that can provide more adsorption sites for contaminants. Adsorption experiments showed that the flower-like Fe-C@C adsorbent exhibited high adsorption capacity (90.14 mg/g) and fast removal of methylene blue (MB). Moreover, the magnetic properties of the nanoparticles, with saturation magnetization of 36.544 emu/g, facilitated their magnetic separation from wastewater. Therefore, the novel flower-like Fe-C@C nanoparticles with integrated adsorptive and magnetic properties have the potential to be an effective adsorbent in dye wastewater treatment.

## 1. Introduction

In recent years, organic dyes have been widely used in the industrial production of paper, textiles, printing, plastics, and other industries [1,2]. Most organic dyes are toxic, even carcinogenic, and are also stable and resistant to photolysis and biodegradation [3,4]. Thus, the development of a convenient and effective technology for treating wastewater contaminated with organic dyes is important. Compared with the decomposition of organic pollutants in water, it is more efficient to separate pollutants via adsorption [5]. Because of their low cost, resistance to acid and alkali corrosion, high specific surface area, and enhanced adsorption capacity, carbon materials have been widely used as adsorbents in the treatment of dye wastewater [6]. However, carbonaceous adsorbents are hard to separate from the dye wastewater after the adsorption process, especially nanoscale adsorbents [7]. A new type of adsorbent, carbon-encapsulated magnetic nanoparticles (CEMNPs), can solve this problem by virtue of its unique core-shell structure, good stability and compatibility, and especially excellent magnetic properties [8,9,10]. However, some problems still remain with CEMNP adsorbents, including the incomplete carbon-encapsulated shells that cause their stability and magnetic properties to degrade under harsh conditions, and a low specific surface area that results in poor adsorption capacity and rate. In addition, obtaining highly purified CEMNPs is still a challenge due to the limitations of the synthesis process.

To solve these problems, several improved solid-state synthesis methods, such as template methods and carbothermal reduction methods [11,12,13], have been used to synthesize CEMNPs. Unfortunately, these methods inevitably produce large amounts of amorphous carbon as a by-product, and some incompletely coated particles still exist. Pulsed laser irradiation can be used to form complete carbon encapsulation layers of CEMNPs [14], but the yield of this process is relatively low and the particle size is difficult to control. In contrast, the particle size of CEMNPs synthesized using spray pyrolysis is relatively uniform [15]. However, agglomeration of CEMNPs at high temperatures may cause a decrease in specific surface area and adsorption activity. Among the methods for synthesizing CEMNPs, chemical vapor deposition (CVD) has received increased attention because of its relatively low cost, potentially high yield, simplicity, and the availability of raw materials [16,17]. However, ceramic materials (e.g., Al_2_O_3_, SiC, and MgO) are often employed as the catalytic supporter in synthesizing CEMNPs using CVD [18,19]. These ceramic supporters are difficult to completely remove from the final product, which affects the purity, and thus degrades the performance of the CEMNPs. Recently, we proposed a novel approach about the synthesis of high-purity carbon-encapsulated cobalt (Co@C) nanoparticles using the CVD method [20]. However, there are still some disadvantages that limit the application of Co@C as an adsorbent, including low saturation magnetization, which makes it difficult to separate the adsorbent from the wastewater. In addition, Co has heavy-metal toxicity and is a potential threat to human health if used in water treatment processes [21]. Therefore, the fabrication of nontoxic high-purity CEMNPs with excellent adsorptive and magnetic properties continues to be a challenge.

In this paper, we present our proposed novel approach to synthesize carbon-encapsulated Fe-C (Fe-C@C) nanoparticles using CVD combined with freeze-drying. Basically, ferric citrate (FeC_6_H_5_O_7_) is loaded onto the surface of sodium chloride (NaCl) by freeze-drying to obtain the Fe_2_O_3_/NaCl catalyst precursor after calcination, which is then used to synthesize Fe-C@C nanoparticles supported on NaCl via CVD method. Because NaCl is water-soluble, the NaCl supporter can be removed by simple washing with water and centrifugation to obtain high-purity Fe-C@C nanoparticles. The synthesized core-shell Fe-C@C nanoparticles have a divergently flower-like morphology and a high specific surface area, which provide more adsorption sites for contaminants. Therefore, they exhibit a high adsorption capacity and fast removal of methylene blue (MB). In addition, because the Fe-C@C nanoparticles have superior magnetic properties, it is easy to separate them from wastewater. Thus, the novel adsorbent Fe-C@C nanoparticles, with their excellent adsorptive and magnetic properties, have great potential for use in the field of water treatment.

## 2. Materials and Methods 

### 2.1. Materials

All chemicals were commercially available and analytical grade unless otherwise stated. FeC_6_H_5_O_7_**·**5H_2_O (99.5% purity) and NaCl (99.5% purity) were obtained from Tianjin Guangfu Fine Chemical Research Institute (Tianjin, China). High-purity argon (Ar, 99.999% purity), high-purity hydrogen (H_2_, 99.999% purity), and acetylene (C_2_H_2_, 99.9% purity) were purchased from Tianjin Liufang Industrial Gas Co., Ltd. (Tianjin, China). MB (C_16_H_18_ClN_3_S**·**3H_2_O, 98.5% purity), an organic dye, was purchased from Tianjin Benchmark Chemical Reagent Co., Ltd. (Tianjin, China).

### 2.2. Synthesis of Fe-C@C Nanoparticles

**Figure 1:**
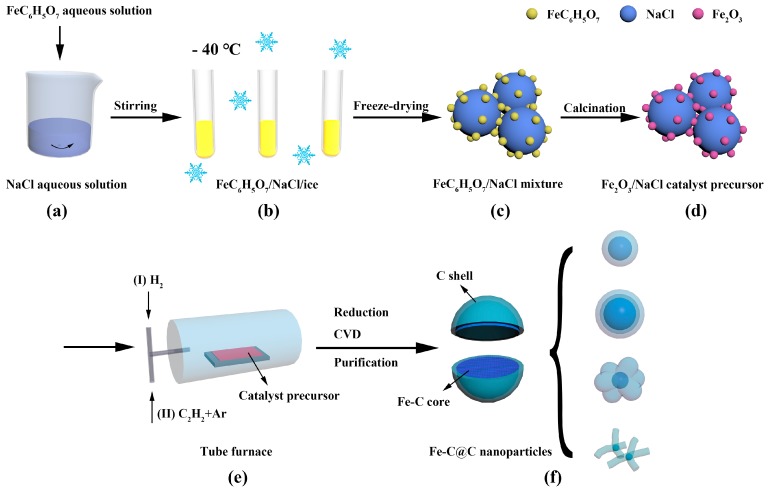
Illustration of the fabrication of Fe-C@C nanoparticles. (**a**) Preparation of FeC_6_H_5_O_7_/NaCl mixed solution. (**b**) Formation of FeC_6_H_5_O_7_/NaCl/ice mixture. (**c**) FeC_6_H_5_O_7_/NaCl mixture obtained via freeze-drying. (**d**) Formation of Fe_2_O_3_/NaCl catalyst precursor. (**e**) Synthesis of Fe-C@C nanoparticles via CVD. (**f**) Fe-C@C nanoparticles with adjustable structures.

Figure 1 presents the process used in this study to fabricate Fe-C@C nanoparticles. Fe and C_2_H_2_ were used as graphitization catalyst and carbon source, respectively. The Fe_2_O_3_/NaCl catalyst precursor was prepared via freeze-drying and calcination using FeC_6_H_5_O_7_**·**5H_2_O and NaCl as raw materials. In a typical procedure, NaCl was dissolved in deionized water with continuous stirring for 30 min to form a NaCl aqueous solution with a mass concentration of 5.97 wt %. A FeC_6_H_5_O_7_**·**5H_2_O aqueous solution with a mass concentration of 1.96 wt % was then added into the NaCl aqueous solution and stirred for 3 h at 60 °C to obtain a uniform mixture with a Fe:NaCl weight ratio of 1:19 (Figure 1a). The mixed solution was transferred to several centrifuge tubes for freeze-drying at −40 °C for 36 h (Figure 1b) to obtain the yellow mixture of FeC_6_H_5_O_7_/NaCl (Figure 1c). The mixture was ground in an agate mortar to a fine powder and then calcined in an air atmosphere at 500 °C for 1 h to obtain the Fe_2_O_3_/NaCl catalyst precursor (Figure 1d). The catalyst precursor was then loaded into an alumina boat, which was placed in the flat-temperature zone of a tube furnace and heated to 600 °C with Ar protection (100 mL/min) at 10 °C/min. When the temperature reached 600 °C, Ar flow was stopped and H_2_ (100 mL/min, Figure 1eI) was introduced into the furnace for 1 h to reduce Fe_2_O_3_, after which the furnace was cooled to one of four synthesis temperatures (300, 350, 400, and 450 °C). When a synthesis temperature was reached, H_2_ was stopped and a gas mixture of C_2_H_2_ (20 mL/min) and Ar (200 mL/min) (Figure 1eII) was immediately introduced into the furnace to synthesize Fe-C@C nanoparticles for 60 min using CVD. Finally, Fe-C@C nanoparticles (Figure 1f) supported on NaCl were obtained after the reactor was cooled to room temperature under an Ar atmosphere (100 mL/min). The Fe-C@C nanoparticles synthesized at 300, 350, 400, and 450 °C were indexed as Samples 1, 2, 3, and 4, respectively.

### 2.3. Purification of Fe-C@C Nanoparticles

First, the synthesized black Fe-C@C nanoparticle powders supported on NaCl were washed by sonication in distilled water for 30 min. Then, the sonicated liquid was placed in centrifuge tubes and centrifuged at 10,000 rpm for 10 min. Finally, a plastic dropper was used to remove the liquid phase containing NaCl in the centrifuge tubes and the precipitate was collected and subsequently dried in a vacuum drying oven at 60 °C for 12 h to obtain the purified Fe-C@C nanoparticles.

### 2.4. Adsorption Experiments

To investigate the adsorptive performance of the synthesized Fe-C@C nanoparticles, adsorption experiments were carried out to measure their adsorption of MB. For adsorption isotherm experiments, Fe-C@C nanoparticles (10 mg) were added to 10-mL MB aqueous solutions of various concentrations (10, 20, 30, 50, 75, 100, 150, 200, 250, and 300 mg/L) to form suspensions. After the suspensions were sonicated for 1 min and oscillated in a shaker at 80 rpm for 24 h, the supernatants were separated by centrifugation at 10,000 rpm for 10 min. The concentrations of MB in the supernatants were determined using ultraviolet–visible (UV–Vis) spectroscopy by measuring the absorbance at 664 nm (characteristic absorbance of MB) relative to a calibration curve recorded under the same conditions. The adsorption capacity of Fe-C@C nanoparticles for MB at equilibrium was calculated by Equation (1).

(1) $$q_{e}=\frac{(c_{0}-c_{e})V}{m}$$

where *q*_e_ is the equilibrium adsorption capacity (mg/g); *c*_0_ and *c*_e_ are the initial and equilibrium concentrations (mg/L) of MB, respectively; *V* is the volume (L) of the MB aqueous solution; and *m* is the mass (g) of Fe-C@C nanoparticles in the suspension. For the removal rate experiments, suspensions formed by adding Fe-C@C nanoparticles (10 mg) to a MB aqueous solution (concentration = 50 mg/L) were sonicated for 1 min and then oscillated for 5, 10, 15, 20, 25, 30, 40, 50 or 60 min. The supernatants were separated by centrifugation at 10,000 rpm for 10 min, and their MB concentrations were determined as in the adsorption isotherm experiments.

### 2.5. Characterization

X-ray diffraction (XRD; D/Max-2500, Rigaku Co., Tokyo, Japan) patterns were recorded to analyze the phase composition of the synthetic products within the angle range of 20°–90° (2*θ*) in steps of 0.02°. The morphologies and microstructures of the powder samples were observed using field emission scanning electron microscopy (FESEM; Nova Nano SEM450, FEI, Hillsboro, OR, USA) and high-resolution transmission electron microscopy (HRTEM; Tecnai G2 F20, FEI, Hillsboro, OR, USA). To evaluate the thermal-oxidative stability and carbon content of the samples, thermogravimetric analysis-differential scanning calorimetry (TGA-DSC; SDT Q600 TGA, TA Instruments, New Castle, DE, USA) was performed in an air atmosphere from 25 to 800 °C with a heating rate of 10 °C/min. To evaluate the crystallinity of the carbon layers, Raman spectra were obtained using a DXR Raman microscope (Thermo Fisher Scientific, Waltham, MA, USA) equipped with an Ar^+^ laser with a 1064-nm line as the excitation source. A vibrating sample magnetometer (7407 VSM, Lake Shore Cryotronics, Westerville, OH, USA) was used to characterize the magnetic properties of the Fe-C@C nanoparticles at 25 °C. N_2_ adsorption–desorption isotherms were measured at −196 °C using an adsorption instrument (ASAP 2020M+C, Micromeritics Instruments Co., Norcross, GA, USA), and the specific surface area, pore volume, and pore size were calculated using the Brunauer–Emmett–Teller (BET) and Barret–Joyner–Halender (BJH) methods [22,23]. UV–Vis spectra were obtained using a UV–Vis spectrophotometer (U-3900H, Hitachi High-Technologies, Fukuoka, Japan) at a fixed wavelength of 664 nm to analyze the adsorptive performance of the Fe-C@C nanoparticles.

## 3. Results and Discussion

### 3.1. Morphology and Structure of Fe-C@C Nanoparticles

**Figure 2:**
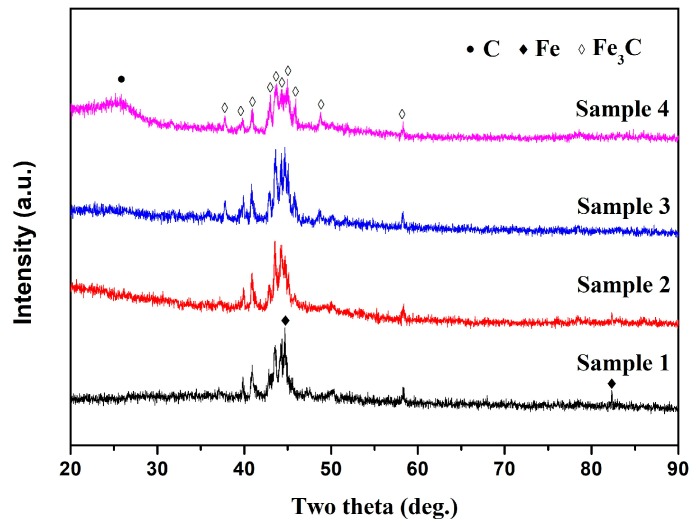
X-ray diffraction (XRD) patterns of purified Fe-C@C nanoparticles synthesized at different temperatures: Sample 1 (300 °C), Sample 2 (350 °C), Sample 3 (400 °C), and Sample 4 (450 °C).

Figure 2 shows the XRD patterns of purified Fe-C@C nanoparticles synthesized at different temperatures. No obvious diffraction peak belonging to the NaCl crystal is seen in the XRD pattern of any of the samples, inferring the complete removal of the NaCl supporter by simple washing and centrifugation. For Samples 1, the diffraction peaks at 2*θ* = 44.7° and 82.3° can be indexed to the (110) and (211) planes of Fe with a body-centered cubic structure (ICDD Card, no. 89-7194), respectively. The other low diffraction peaks can be indexed to the Fe_3_C phase (ICDD Card, no. 76-1877) based on their positions and relative intensities. The characteristic peaks belonging to the crystalline graphite phase are not observed because of the low content and crystallinity of the deposited graphite phase in these samples, which is consistent with the literature [20,24]. For Sample 2, the diffraction peaks of Fe at 44.7° and 82.3° become weakened obviously. With a growth temperature of 400 °C (Sample 3), the diffraction peaks of Fe disappear and only the characteristic peaks of Fe_3_C remain. Furthermore, compared with the XRD patterns of Samples 1 and 2, that of Sample 3 has more low-intensity diffraction peaks at 2*θ* = 37.8°, 46.0°, and 48.7°, which can be associated with the (021), (211), and (113) planes of Fe_3_C, respectively, confirming that Sample 3 is mainly composed of Fe_3_C. Similar to Samples 1 and 2, the characteristic peaks belonging to the crystalline graphite phase are still not observed. The diffraction peaks of Fe_3_C for Sample 4, synthesized at 450 °C, are similar to those of Sample 3, except for the appearance of a broad halo peak around 2*θ* = 26.5°, which can be associated with the (002) plane of graphite. This broadened diffraction peak suggests that the synthesized carbon products in Sample 4 have more structural defects than the crystalline graphite. The XRD results in Figure 2 show that the metallic phase in the synthesized Fe-C@C nanoparticles transforms from Fe/Fe_3_C phases in Samples 1 to a simple Fe_3_C phase in Samples 2, 3, and 4. This transformation can be attributed to the high reactivity between Fe and C atoms at higher synthesis temperatures [25].

**Figure 3:**
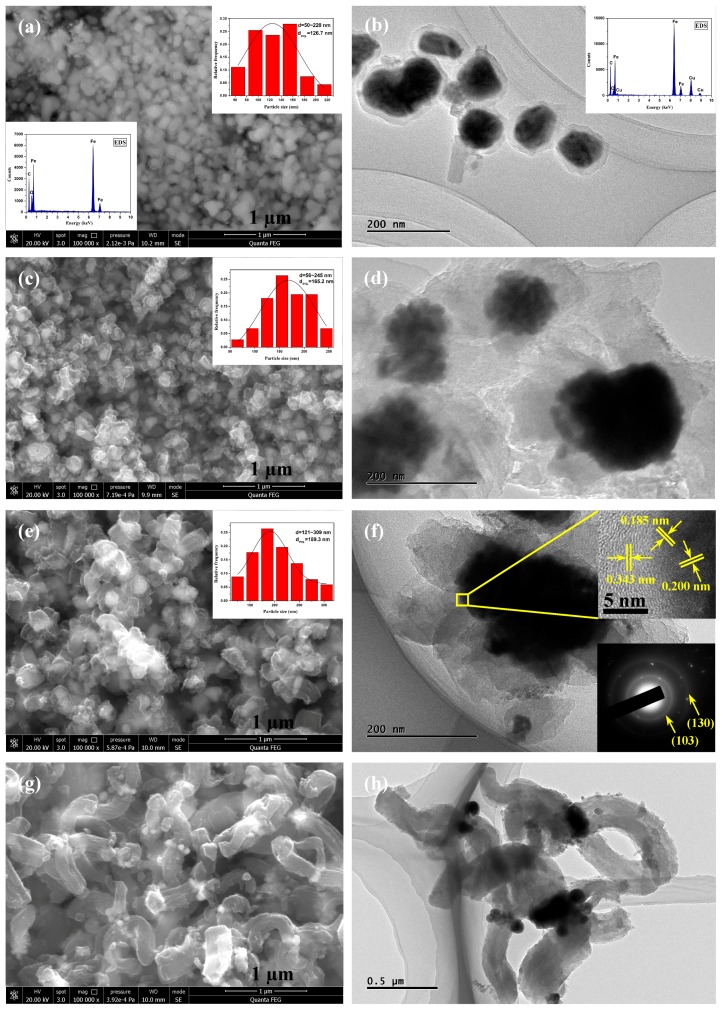
Scanning electron microscopy (SEM) and transmission electron microscopy (TEM) images of Fe-C@C nanoparticles: (**a**,**b**) Sample 1, (**c**,**d**) Sample 2, (**e**,**f**) Sample 3, and (**g**,**h**) Sample 4 (Inset of a: the energy-dispersive X-ray spectroscopy (EDS) analysis and particle size distribution based on SEM; inset of b: the EDS analysis based on TEM; insets of c and e: the particle size distribution; inset of f: HRTEM image of the square region and the diffraction pattern).

The SEM and TEM images in Figure 3 show the morphology and microstructure of the Fe-C@C nanoparticles synthesized at different temperatures. The morphology and microstructure of the synthesized Fe-C@C nanoparticles vary with the synthesis temperature, and there are no bare Fe-C nanoparticles, indicating that the carbon encapsulation layers were successfully deposited onto the surfaces of the Fe catalysts. Figure 3a shows that the Fe-C@C nanoparticles in Sample 1, synthesized at 300 °C, are quasi-spherical. According to the particle size distribution (inset of Figure 3a) based on the SEM analysis, the diameters of the nanoparticles vary from 50 to 220 nm, with an average diameter of 126.7 nm. The energy-dispersive X-ray spectroscopy (EDS) image as an inset of Figure 3a shows that the main elements of Sample 1 are C, Fe, and O without the existence of Cl and Na, further indicating that the NaCl supporter was completely removed from the Fe-C@C nanoparticles. Figure 3b shows that the synthesized Fe-C@C nanoparticles of Sample 1 have a typical core-shell structure, with Fe-C cores and amorphous carbon shells. The carbon shells encapsulating the Fe-C cores are thin (approximately 20 nm thick) due to the low catalytic activity of the Fe nanoparticles at the low synthesis temperature [26]. The EDS analysis inserted in Figure 3b shows that the main elements of Sample 1 are Fe, C, and Cu coming from the Cu TEM grid. Figure 3c shows that Sample 2, synthesized at 350 °C, exhibits a rough and irregular particle shape compared with Sample 1 (Figure 3a) and has a larger average diameter (165.2 nm) than Sample 1. The larger diameter results from the accelerated decomposition of the carbon source caused by the enhanced catalytic activity of Fe catalyst nanoparticles and the reactivity of Fe and C atoms at the higher synthesis temperature [27,28]. Figure 3d also shows the rough and irregular particle shape of Sample 2, and the synthesized Fe-C@C nanoparticles have a thicker carbon shell (approximately 70 nm) compared with that of Sample 1. Figure 3e shows that the Fe-C@C nanoparticles of Sample 3, synthesized at 400 °C, exhibit carbon shells diverging in different directions. The particle size distribution (inset of Figure 3e) shows that the diameters of Sample 3 nanoparticles are in the range of 121–309 nm, with an average diameter of 189.3 nm. The TEM image in Figure 3f further shows that the Fe-C@C nanoparticles have a “flower-like” structure, that is, external rough and divergent carbon shells and internal metal cores. These flower-like Fe-C@C nanoparticles have coarse surfaces and many porous channels, and thus, they can provide more adsorption sites for contaminants, which help improving the adsorption capacity of the nanoparticles when used as adsorbents in treating dye wastewater. The HRTEM image in the upper inset of Figure 3f shows that the Fe-C particles are closely connected to the carbon-encapsulated layers, and their lattice fringes can be clearly observed. The interplanar spacing of 0.185 and 0.200 nm can be indexed to the (122) and (103) planes of Fe_3_C, respectively [25]. The carbon-encapsulated layers maintain the state of short-range order (with an interplanar spacing of 0.343 nm) and long-range disorder, like amorphous carbon. The selected area electron diffraction (SAED) pattern in the lower inset of Figure 3f shows two diffraction rings that are attributed to the (103) and (130) planes of Fe_3_C, respectively, further confirming that the Fe-C cores are composed of polycrystalline Fe_3_C. When the synthesis temperature is 450 °C for Sample 4, short and thick carbon nanofibers (CNFs), rather than Fe-C@C nanoparticles, are synthesized, as shown in Figure 3g,h, because of the enhanced catalytic activity of the Fe catalysts [29], which reduces the purity of the synthesized Fe-C@C nanoparticles.

**Figure 4:**
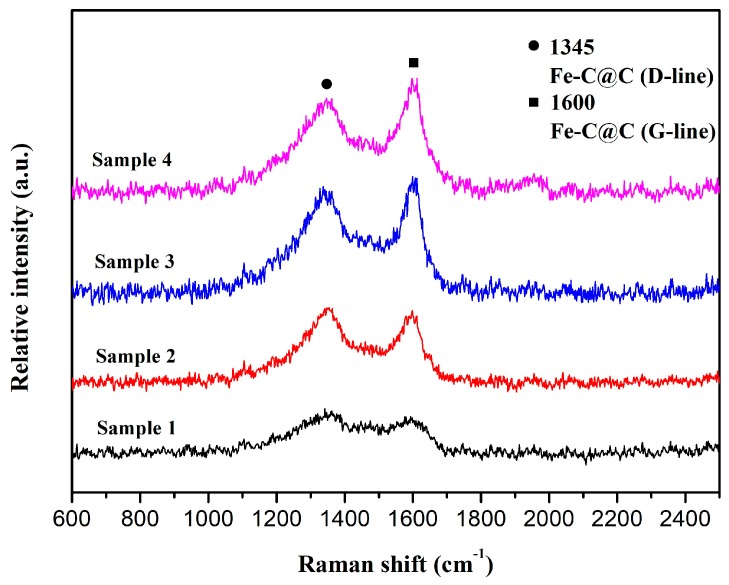
Raman spectra of Fe-C@C nanoparticles.

Figure 4 shows the Raman spectra obtained to evaluate the degree of graphitization of the Fe-C@C nanoparticles synthesized at different temperatures. All samples have two Raman bands at ~1345 cm^−1^ (D band) and ~1600 cm^−1^ (G band), which are associated with the vibrations of carbon atoms with dangling bonds for the in-plane terminations of disordered graphite (D) and the vibrations in sp^2^-bonded carbon atoms in a 2D hexagonal lattice (G), respectively [30]. The relative intensity *I*_D_/*I*_G_ of Samples 1, 2, 3, and 4 is calculated to be 1.12, 1.05, 0.97, and 0.95, respectively. Although the degree of graphitization of the Fe-C@C nanoparticles increases with increasing synthesis temperature, the values of *I*_D_/*I*_G_ for all samples are larger than that of graphite carbon, indicating the existence of many structural defects in the synthesized carbon shells. These defects are conducive to forming abundant porous structures that improve the adsorption property of the Fe-C@C nanoparticles.

**Figure 5:**
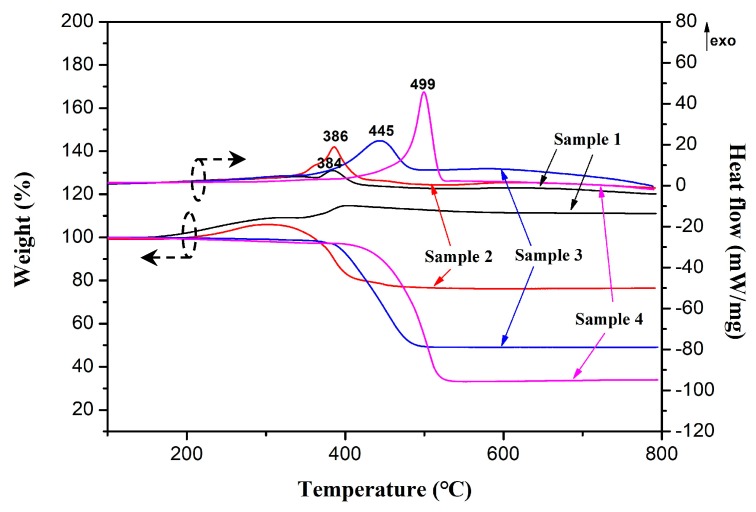
Thermogravimetric analysis-differential scanning calorimetry (TGA-DSC) curves of Fe-C@C nanoparticles.

Figure 5 shows the TGA-DSC curves of the Fe-C@C nanoparticles synthesized at different temperatures. During the heating process, all the samples exhibit similar exothermic behavior at different oxidation temperatures, which is associated with various changes in sample weight. The weight increase in Sample 1 at the heating stage from 152 to 402 °C is due to the oxidation of iron nanoparticles to iron oxide. An oxidation exothermic peak appears at 384 °C with an increase in weight of approximately 15.2 wt %. This is the comprehensive effect of the oxidation of carbon encapsulation layers and Fe-C cores, that is, the conversion of carbon to carbon dioxide leading to a decrease in weight, the formation of iron oxide causing an increase in weight, and the oxidations of carbon and iron are all part of the exothermic reaction. Similarly, Sample 2 exhibits a slight weight increase at the initial heating stage from 192 to 304 °C. On the other hand, Sample 2 losts weight between 304 and 470 °C because of its higher carbon content. Approximately 76.2 wt % of the sample remains after the heating process. An oxidation exothermic peak appears at approximately 386 °C. Sample 3 has only a single-step weight loss and stops losing weight near 500 °C. The oxidation exothermic peak shifts right to approximately 445 °C, indicating the enhanced thermal-oxidative stability of the Fe-C@C nanoparticles. Approximately 49.3 wt % of Sample 3 remains after performing the TGA. The weight loss for Sample 4 is obviously greater than that of the other samples, and only approximately 33.4 wt % of the sample remains after TGA analysis, indicating a higher carbon content. The sample stops losing weight near 536 °C. The oxidation exothermic peak at approximately 499 °C indicates the higher thermal-oxidative stability of Sample 4.

**Figure 6:**
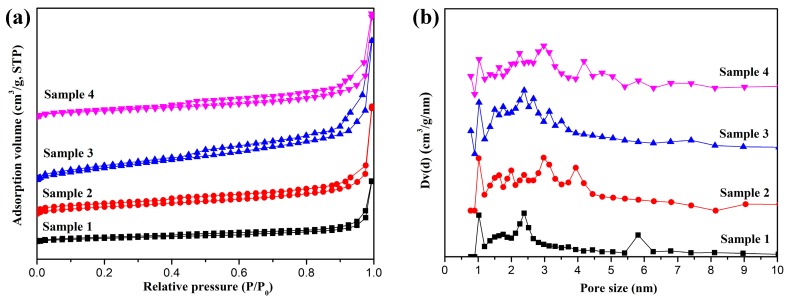
(**a**) N_2_ adsorption-desorption isotherms and (**b**) pore size distributions of Fe-C@C nanoparticles.

Figure 6 and Table 1 show the N_2_ adsorption-desorption isotherms, pore size distributions, and textural parameters of the synthesized Fe-C@C nanoparticles. The isotherms in Figure 6a all exhibit hysteresis loops from relative pressure 0.4 to 1.0 because of the irreversibility between the adsorption and desorption processes. Because N_2_ fills the spaces in the synthesized Fe-C@C nanoparticles, the amount of adsorption increases dramatically as the relative pressure approaches 1.0 [31]. The N_2_ adsorption-desorption isotherms of Samples 1 and 2 exhibit two narrow hysteresis loops, that is, the close intervals between adsorption and desorption branches, indicating that there are fewer pores in the synthesized samples. Accordingly, Figure 6b shows that Samples 1 and 2 have only weak peaks that are attributed to the clearance of carbon layers, and Table 1 shows that their specific surface areas and pore volumes are smaller than those of Samples 3 and 4. According to the IUPAC classification [32,33], the isotherms of Samples 3 and 4 can be classified as typical type IV curves with protuberant hysteresis loops, indicating the coexistence of micropore and mesopore in the materials [34,35]. In Figure 6b, Sample 3 exhibits a broad peak ranging from 1.5 to 4.0 nm and centers at approximately 2.3 nm, inferring that many pores are distributed in the outer carbon layer. The specific surface area and pore volume of Sample 3 are calculated to be 169.4 m^2^/g and 0.50 cm^3^/g, respectively, as shown in Table 1. The pore size distribution of Sample 4 is similar to that of Sample 3, but its specific surface area (124.7 m^2^/g) and pore volume (0.44 cm^3^/g) are lower. From the above experimental results, it can be concluded that Sample 3 possesses the high specific surface area and pore volume because of its “flower-like” structure. The porous characteristic provides a large number of adsorption sites for pollutants and is conducive to enhancing the adsorption capacity and removal rate of the Fe-C@C nanoparticles.

### 3.2. Magnetic Properties

**Figure 7:**
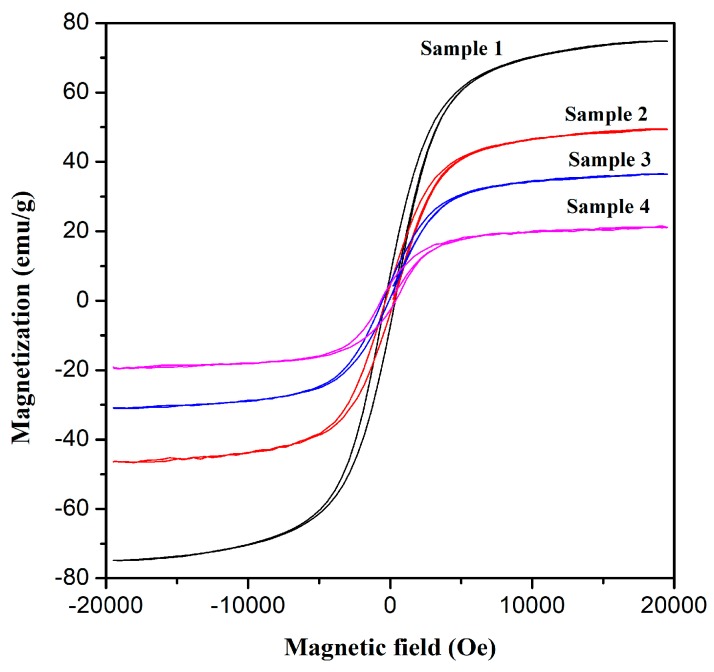
Hysteresis loops of Fe-C@C nanoparticles.

It is very important to evaluate the magnetic properties of the synthesized Fe-C@C nanoparticles to separate the adsorbents from wastewater. Table 2 and Figure 7 present the magnetic parameters and hysteresis loops of the Fe-C@C nanoparticles, respectively. The saturation magnetization (*M*_s_) and coercive force (*H*_c_) of the synthesized Fe-C@C nanoparticles are in the range of 21.533–74.793 emu/g and 358.83–621.33 Oe, respectively, which are all higher than the reported values of Fe-C@C nanoparticles with similar composition [11,36]. The ratios of the remanence to saturation magnetization (*M*_r_/*M*_s_) are in the range of 0.102–0.212, which indicates the improved ferromagnetism of the Fe-C@C nanoparticles. The magnetic properties of the Fe-C@C nanoparticles synthesized at different temperatures vary with synthesis temperature. Figure 7 shows that the magnetization of the synthesized Fe-C@C nanoparticles yields square hysteresis loops with periodically varying magnetic fields. However, the squareness of the hysteresis loops is compressed as the synthesis temperature increases, indicating a gradual reduction in *M*_s_, which is in accordance with the magnetic parameters in Table 2. In addition, the *M*_s_ of Samples 1–4 are all lower than that of bulk Fe (218 emu/g) and bulk Fe_3_C (140 emu/g) [37,38]. This decrease in *M*_s_ can be attributed to the carbon layers that encapsulate the Fe-C cores. Carbon is a diamagnetic substance that weakens the *M*_s_ of Fe-C@C nanoparticles [39,40]. As the synthesis temperature increases, the content of diamagnetic carbon in the Fe-C@C nanoparticles increases, resulting in a further decrease in *M*_s_. Furthermore, the transformation of Fe in Samples 1 and 2 to Fe_3_C in Samples 3 and 4 also causes the decrease in *M*_s_. In addition, the *H*_c_ of the Fe-C@C nanoparticles increases with the increase in the synthesis temperature, as seen in Table 2. This can be attributed to the weakened dipole interaction owing to the thickened carbon layers deposited on the Fe-C cores [41]. The *H*_c_ of the synthesized Fe-C@C nanoparticles is much greater than that of pure bulk Fe (~1 Oe) or Fe_3_C (usually < 100 Oe). This is due to the effect of the nanoscale Fe-C cores resulting in a high *H*_c_ [42]. As a result, the synthesized Fe-C@C nanoparticles have improved ferromagnetism that allows them to quickly respond to an external magnetic field, which is beneficial in separating them from wastewater.

### 3.3. Adsorption Properties

**Figure 8:**
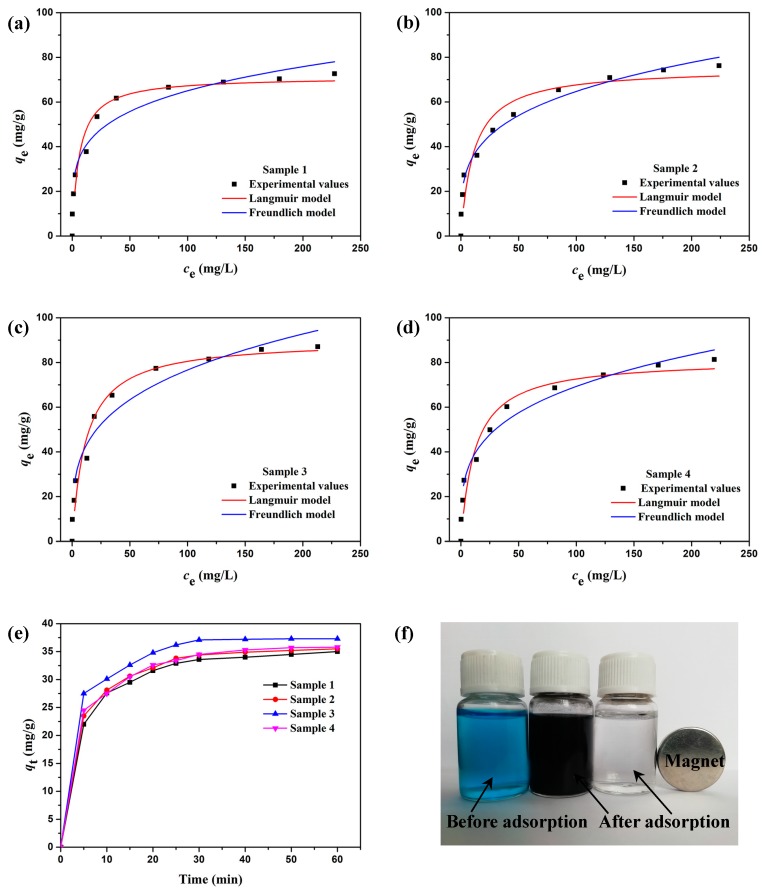
(**a**–**d**) Experimental values of *c*_e_ and *q*_e_ obtained by adsorption isotherm experiments and the fitted curves of Langmuir and Freundlich isotherm models for Samples 1–4. (**e**) Adsorption capacities *q*_t_ of Samples 1–4 measured at various times. (**f**) Photograph of a MB aqueous solution (30 mg/L) before and after adsorption via Sample 3 over 30 min and magnetic separation by an external magnet.

The application of Fe-C@C nanoparticles as an adsorbent in separating pollutant from wastewater was investigated using MB as the pollutant. The adsorption behaviors of Samples 1–4 were described using the Langmuir and Freundlich isotherm models (Equations (2) and (3), respectively) [43,44]. The corresponding parameters are given in Table 3.

(2) $$\frac{c_{e}}{q_{e}}=\frac{1}{K_{L}Q_{\max}}+\frac{c_{e}}{Q_{\max}}$$

(3) $$\ln q_{e}=\ln K_{F}+\frac{1}{n}\ln c_{e}$$

where *c*_e_ (mg/L) is the equilibrium concentration of MB in the aqueous solution, *q*_e_ (mg/g) is the equilibrium adsorption capacity, *K*_L_ (L/mg) is the binding constant of the Langmuir adsorption, *Q*_max_ (mg/g) is the maximum adsorption capacity, *K*_F_ [(mg/g)·(L/mg)^1/*n*^] is the Freundlich constant, and 1/*n* is the Freundlich adsorption intensity parameter. Figure 8a–d present the experimental values of *c*_e_ and *q*_e_ obtained by adsorption isotherm experiments and the fitted curves of the Langmuir and Freundlich isotherm models. The fitted curves of both models match the experimental data well, which indicates the uniform distribution of adsorption sites on the surfaces of the Fe-C@C nanoparticles [31]. The similar correlation coefficients (*R*^2^) in Table 3 are also indicative of the uniform adsorption site distribution. From the Langmuir model, the *Q*_max_ values of Samples 1–4 are 71.28, 75.16, 90.14, and 81.45 mg/g, respectively, as listed in Table 3. Sample 3 has the highest adsorption capacity for MB. The rough and divergent carbon shells of Sample 3, with their porosity, play pivotal roles during the adsorption process via providing more sites for the adsorption of the pollutant, and thus result in a remarkably enhanced adsorption capacity. For the Freundlich model, adsorption is easy when the adsorption intensity 1/*n* is between 0.1 and 1, but becomes more difficult when above 1 [45,46]. As listed in Table 3, 1/*n* is in the range of 0.222–0.275 for Samples 1–4, which suggests that the Fe-C@C nanoparticles easily adsorb MB in the aqueous solution. Figure 8e presents the adsorption capacities (*q*_t_) of Samples 1–4 measured at various times. All the adsorption curves exhibit a similar trend in the change in *q*_t_ and reach equilibrium within 30 min, suggesting fast removal of MB. After adsorption for 30 min, *q*_t_ of Sample 3 is calculated to be 37.2 mg/g when the initial concentration of the MB aqueous solution is 50 mg/L, which is higher than *q*_t_ of Sample 1, 2, and 4, i.e., 33.6, 34.4, and 34.5 mg/g, respectively. Further experiments confirm that Sample 3 has preferable adsorption behavior. Figure 8f shows the MB aqueous solution before and after adsorption via Sample 3 over 30 min and magnetic separation by an external magnet. The original blue MB aqueous solution becomes black after adsorption and removal of MB by Sample 3. Further, the solution becomes clear after the magnetic separation of Fe-C@C nanoparticles via an external magnet. Therefore, the flower-like Fe-C@C nanoparticles, with their excellent adsorptive and magnetic properties, can be used as an efficient adsorbent in treating dye wastewater.

## 4. Conclusions

Novel flower-like Fe-C@C nanoparticles with integrated adsorptive and magnetic properties were successfully fabricated for use as adsorbing materials. Via freeze-drying, the Fe metallic source was loaded on a NaCl supporter, after which the combination was easy purified via simple washing and centrifugation to obtain high-purity Fe-C@C nanoparticles. Using the CVD method, the thickness, morphology, and structure of the carbon layers deposited on the Fe catalyst surfaces were adjusted by changing the synthesis temperature. The optimal Fe-C@C nanoparticles, synthesized at 400 °C, exhibited a divergently “flower-like” morphology, with coarse surfaces and many porous channels. Their Raman spectra revealed a lower degree of graphitization, suggesting the existence of many structural defects in the synthesized carbon shells that help in the formation of abundant porous structures. TGA-DSC analysis showed the enhanced thermal-oxidative stability of the flower-like Fe-C@C nanoparticles. N_2_ adsorption-desorption isotherms confirmed their porosity, with high specific surface area (169.4 m^2^/g) and pore volume (0.50 cm^3^/g), which can provide many adsorption sites for pollutants and can enhance the adsorption capacity and pollutant removal rate. Adsorption experiments showed that the flower-like Fe-C@C nanoparticles possessed high adsorption capacity (90.14 mg/g) and fast removal of the pollutant MB. In addition, the synthesized Fe-C@C nanoparticles exhibited improved ferromagnetism, which facilitates the separation of adsorbents from dye wastewater. With these characteristics, the novel flower-like Fe-C@C nanoparticles have great potential as adsorbents in the treatment of dye wastewater.

## Figures and Tables

**Table 1:** Specific surface area, pore volume, and pore size of Fe-C@C nanoparticles.

Materials	Specific Surface Area (m^2^/g)	Pore Volume (cm^3^/g)	Pore Size (nm)
Sample 1	79.4	0.25	2.37
Sample 2	105.5	0.35	3.14
Sample 3	169.4	0.50	2.18
Sample 4	124.7	0.44	2.83

**Table 2:** Magnetic parameters of Fe-C@C nanoparticles.

Materials	*M*_s_ (emu/g)	*M*_r_ (emu/g)	*M*_r_/*M*_s_	*H*_c_ (Oe)
Sample 1	74.793	7.5955	0.102	358.83
Sample 2	49.532	5.7361	0.116	426.90
Sample 3	36.544	5.6221	0.154	560.61
Sample 4	21.533	4.5716	0.212	621.33

**Table 3:** Langmuir and Freundlich isotherm model parameters.

Materials	Langmuir Model			Freundlich Model		
	*Q*_max_ (mg/g)	*K*_L_ (L/mg)	*R* ^2^	*K*_F_ [(mg/g)·(L/mg)^1/*n*^]	1/*n*	*R* ^2^
Sample 1	71.28	0.167	0.937	23.42	0.222	0.940
Sample 2	75.16	0.090	0.897	19.26	0.263	0.987
Sample 3	90.14	0.083	0.955	21.58	0.275	0.952
Sample 4	81.45	0.083	0.925	20.07	0.269	0.979
